# Reliable but Wrong? Automated Detection of Heart Rate Artifacts as a Source of Bias in Pediatric EEG Data

**DOI:** 10.21203/rs.3.rs-9419796/v1

**Published:** 2026-07-07

**Authors:** Brenna Arledge, Tori Hollen, Akhila K. Nekkanti, Elizabeth A. Skowron, Lauren E. Ethridge, David E. Bard

**Affiliations:** University of Oklahoma; University of Oklahoma; Oregon State Department of Human Services; Pennsylvania State University; University of Oklahoma; University of Oklahoma Health Sciences Center

**Keywords:** Electroencephalography (EEG), Artifact detection, ICLABEL, Pediatric EEG, Cardiac artifact, Independent component analysis (ICA)

## Abstract

Automated processing pipelines are increasing in popularity for analysis of EEG data, however care must be taken when using automated algorithms are applied outside their training dataset. This study assessed whether ICLABEL, a widely used artifact detection algorithm trained primarily on adult EEG, generalizes to pediatric EEG, with a focus on cardiac artifact which is easily identified by trained researchers yet prone to confounding automated detection due to spectral overlap with common EEG outcomes. Resting EEG child data from a randomized controlled trial (n = 47) was analyzed via ICLABEL and compared to manual artifact detection. ICLABEL miscategorized cardiac artifacts 100% of the time, with 66 of 92 components displaying a heart rate waveform labeled as 0% likelihood of cardiac artifact. To assess downstream impact, data with manual artifact removal was compared to data retaining heart rate components, examining effects on theta and beta power and theta/beta ratio, frequencies that overlap with cardiac artifact. Retention of heart rate artifacts significantly elevated theta and beta power estimates and introduced a spurious treatment effect on eyes-closed beta power, underscoring the importance of proper artifact removal before interpreting study outcomes. We recommend supervised use of the algorithm when applied to pediatric EEG or data that diverges from neurotypical adult data.

## Introduction

Electroencephalography (EEG) is a commonly used method for measuring brain activity due to its relatively low cost, mobility, sub-millisecond temporal resolution, and high level of participant tolerance even in complex behavioral conditions. EEG can be collected and analyzed in a number of variations, and a gold standard for EEG collection and processing implementation in research has not been established. One consideration when determining best practice for EEG data collection and analysis is the impact of outside sources of noise, or artifacts. Artifacts refer to non-neural, unwanted signals that can be biological, environmental, or technological in nature. Regardless of source, artifacts impact the neural signal of focus. Artifacts can occur due to technical problems such as faulty EEG nets, behavioral problems such as movement of the participant, physiological factors (i.e., eye blinks, eye movement, heart rate), and environmental noise, all of which are not of interest to most researchers [1, 2]. Although there are safeguards that can be put in place to help prevent artifacts such as notch filters, proper preparation, behavioral coaching and monitoring, and isolated environments, there is no perfect solution for preventing artifacts outright. Thus, artifacts are common in EEG data and require additional analytical consideration to account for them.

In order for EEG data to be usable and accurate to their source, the data must be delicately and deliberately cleaned of artifacts. There are a multitude of viable methods that can be used to remove artifacts, but currently there is no consensus on which methods must be used, leading to a lack of consistency across EEG pre-processing methods. A common source of inconsistency across artifact removal methods stems from a variety of differences in EEG systems, caps, tasks, and availability of software or personnel across studies, which may incur differing levels of artifact [3–5]. (Common methods for artifact removal include regression methods, wavelet transform, blind source separation (BSS), empirical mode decomposition (EMD), and independent component analysis (ICA). The purpose of artifact removal across methods remains the same, but the specific mechanisms behind detecting and removing artifacts varies [3, 4, 6]. Thus, some methods are better at successfully identifying and separating some types of artifacts than others. Regardless of the method chosen, artifact removal requires the use of an automated pipeline, a trained individual manually making decisions, or a combination of the two. With the rise of AI and software as well as the issue of subjectiveness, the automated option has become increasingly more popular.

**Figure 1:**
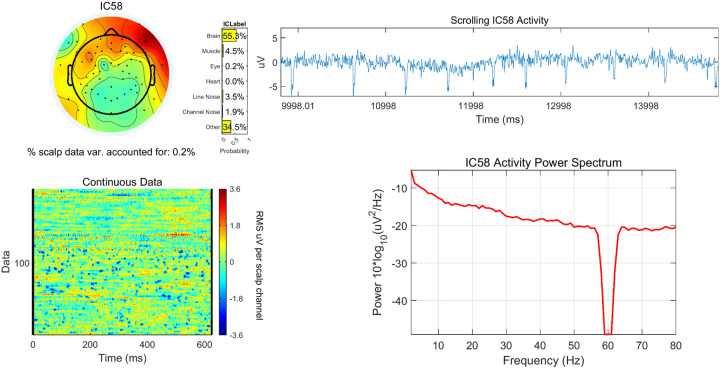
ICA component misrepresenting heart rate as brain. This figure demonstrates an exemplar misclassification of cardiac activity, which can clearly be identified in the upper right panel by its characteristic waveform and the lower left panel by repetitive amplitude fluctuations. Mixing of noise sources and enhanced beta activity from the cardiac artifact itself distorts the power spectrum (lower right) and scalp distribution (upper left), leading ICLABEL to misclassify this component as primarily brain activity with 0% heart artifact (upper middle panel).

Automated pipelines are software applications that perform computations and decision making during some EEG processing steps, commonly guided by specific formulae, machine learning or AI algorithms. The purpose of automated pipelines is threefold: to make some or all of the steps of EEG preprocessing quicker for the trained EEG analyst, to make EEG analysis more straightforward for non-expert or new users, and to improve reliability and standardization across studies. The simplest method for automated artifact rejection is to remove all trials marked as contaminated via implementation of a pre-determined threshold. The threshold marks a maximum in amplitude that is often considered biologically implausible for neural data, typically associated with large scale body movements, eye blinks and saccades, and electrical noise around 60 Hz [7]. This method is well received by many EEG researchers, especially those that record long trials. However, it is common for these methods to result in a drastic loss of usable data [7]. In addition, it is less useful with artifacts of lower amplitude, such as heart rate and muscle artifacts, that still negatively affect the data and outcomes if left unaddressed [7]. Instead, machine learning methods such as ICA that statistically model artifacts and then reconstruct the underlying data, can enable more data retention than wholesale removal of data segments via thresholding. However, ICA requires a decision-making process in which each component is assigned a label of brain activity or artifact, which can either be done manually by a trained human user, or via a number of automated pipelines that use a variety of metrics to categorize. For example, ICLABEL is a project that uses crowdsourcing from a large number of manual users to train its algorithm to identify components using a structured Bayesian framework and multi-level “votes” [8]. Another common decision algorithm, Multiple Artifact Rejection Algorithm (MARA), evaluates ICA components on 6 features (mean local skewness, log alpha power, lambda, fit error, range within pattern, and current density norm) and combines these metrics with manual training data to classify components [9]. One major benefit of automated pipelines is in standardized decision making across users. However, at present, manual cleaning by well-trained users, while inherently subjective, performs more accurately for specific removal of artifacts, particularly in unusual or messy datasets, and in turn saves more data than an automated pipeline, although further model improvement will likely close this gap [5, 10, 11]. In particular, reliable and standardized outputs, while critical for accurate interpretation of EEG across studies, must not simply be reliable. In some cases, automated pipelines can be found to make reliably inaccurate classifications. For example, most automated pipelines and decision algorithms have been trained on healthy adult EEG data, which differs both quantitatively and qualitatively from typically developing pediatric, neurodevelopmental disorder, and psychiatric adult data [12, 13]. This can lead to mislabeling of components that are typical of a special population’s brain activity because they do not “fit” the trained model criteria. Despite the technical paper for ICLABEL, utilizing adult heart rate identification as a key performance indicator to highlight the excellent classification performance of this algorithm, this commonly used EEGLab plugin falls prey to the same training limitations and thus may underperform on identifying key artifacts like heart rate in data that are not well represented in the training dataset [8]. When artifacts are retained in the data due to inaccurate labeling by the automated pipeline, data, results, and interpretations can be significantly impacted. This is particularly critical when new users are working with automated pipelines to implement EEG analyses. Ostensibly, automated decisions should mimic the best trained experts and outperform a new user. However, when these pipelines fail, new users may not yet have enough experience to recognize or identify those failures. As such, artifacts can easily be misinterpreted as cognitive processes or biomarkers of pathological activity, which will bias interpretations and diagnoses [4]. Cardiac artifacts like heart rate are a good example of intrinsic artifacts that are low amplitude. Thus, they may not be removed by amplitude thresholding and consist of frequencies that overlap with commonly analyzed EEG rhythms, namely delta, theta, and beta activity, which can make frequency-based thresholding difficult [5, 14]. As a result, heart rate can be easily confused with EEG rhythms, introducing confounds across time and frequency domains [15]. However, a trained researcher can easily spot the characteristic waveform for cardiac activity using pattern recognition and identify it as an artifact. This combination of threshold-based classification failure versus clear visual classification makes heart rate an ideal artifact category with which to test the accuracy of training for automated artifact detection algorithms against manual detection. Figure 1 presents an example of mis-identified heart rate as brain, in which a trained professional would be able to detect, but not an automated pipeline.

Although heart rate artifacts can impact all data, they can be particularly impactful on EEG metrics consisting of the frequency ranges in which they overlap, of which one common example is the EEG measure of theta-beta ratio (TBR). TBR represents the relative proportion of theta and beta power in an EEG dataset. Heart rate artifacts overlap with high-delta and theta frequencies, which can artificially inflate theta estimates, but at the same time the non-sinusoidal shape of the heart rate waveform, leads to the artifact being represented in higher harmonics of the initial band, which can artificially inflate beta power estimates, which may or may not be linearly related to its impact on theta estimates [16]. TBR is thought to reflect activation, attentional capacity, and cognitive processing and is operationalized as the ratio of theta to beta activity in EEG activity [17, 18]. A higher TBR indicates an increase in theta and decrease in beta power, which have been attributed to difficulties in attention and cognitive control [19]. In addition, higher theta/beta profiles have been observed in children exposed to high levels of psychosocial risk [20].

Figure 1.

The purpose of the current study was to examine the performance of ICLABEL, an automated artifact detection algorithm trained primarily on adult EEG, in a sample of child welfare-involved children. Cardiac artifact served as the exemplar given its straightforward identifiability by trained researchers and its spectral overlap with EEG outcomes of interest, specifically theta and beta power and theta/beta ratio.

Given that this pediatric, high-adversity sample likely diverges from ICLABEĽs adult training data, we sought to evaluate both the algorithm's artifact detection accuracy and the downstream impact of misclassified cardiac components on TBR outcomes.

## Results

Descriptives for TBRs, theta and beta power per PCIT group for both data with heart rate retained and data with heart rate removed are shown in Supplementary Table S1. Out of 47 participants, 14 had at least one heart rate component in their pre-intervention data and 19 had at least one in their post intervention data, totaling 27 participants.

Next, we calculated effect sizes for the difference in means between heart rate retained and heart rate removed data, for the EEG variables described in Supplementary Table S1. Effect sizes (Cohen’s d) with 95% confidence intervals for TBR and theta and beta relative power are shown in Table 1. Overall, effect sizes for heart rate artifact on TBR were small or medium and did not differ significantly from zero. This is understandable given that the ratios are, by nature, standardized and thus would be less vulnerable to bias, assuming linear effects of artifact on the inputs to the ratio calculation. For both theta and beta power, effect sizes were much larger, indicating that power values were much more impacted by heart rate artifact, although given the non-significant results for the ratio between the two values, and the similar effect sizes within each group for theta and beta power effects, the bias introduced by heart rate artifact on these power values is similar across frequency bands and largely negated by calculation of the theta/beta ratio. Nonetheless, power values themselves are frequently analyzed as stand-alone values, and conclusions based on these values could be impacted by heart rate retention. ACE and CHAOS scores remain the same for all subsequent analysis, refer to Supplementary Table S2.

Importantly, these effect sizes represent the impact of heart rate on a dataset in which less than half of the datasets contained a heart rate component, with manual identification and removal of all heart rate components for the heart rate removed condition. This means that for participants with no identifiable heart rate component, both heart rate retained, and heart rate removed datasets are identical, and temper effects of heart rate on group means. To examine the extent to which heart rate could theoretically impact EEG data during automated cleaning, we next investigated two questions: the frequency with which ICLABEL mislabels a heart rate component, and the impact of heart rate on group means when only participants with at least one heart rate component are utilized in the analyses.

**Figure 2:**
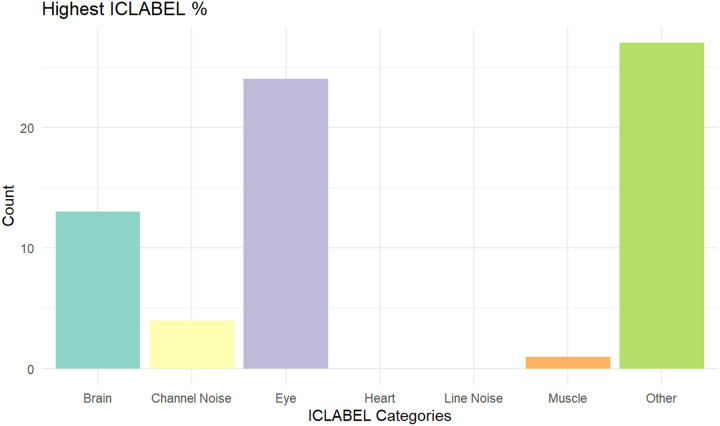
Count of ICLABEL’s misidentification of heart rate components (n=92) Blue bars refer to ICA components labeled as Brain, Yellow as Channel noise, Purple as Eye, Orange as muscle, and Green as other.

Across the subset of individuals with at least one heart rate component, 92 individual components with characteristics consistent with heart rate were identified (Fig. 2). ICLABEL includes 7 possible categories for labeling each component, with the last category being “other” for all components not meeting specific matching criteria for any other category. Each component labeling output includes a percent contribution of each category to that component, totaling 100%. Figure 2 depicts the distribution of categorization for the label with the highest contribution for each component identified manually as heart rate. Importantly, t no components were labeled as primarily heart rate. ICLABEL contribution percentages attributed to the heart category were less than 5% for all components analyzed, with 66 of 92 components labeled 0% heart rate. Even more concerning are the 13 components labeled as primarily brain activity. If a user were to remove components using an automated pipeline based solely on ICLABEL categorizations for heart rate, this artifact would be retained 100% of the time, causing concern for the use of ICLABEL without additional supervision.

Given the severity of the misidentification of heart rate components by ICLABEL, we felt justified in analyzing the “worst-case scenario”, in which every participant has at least one heart rate component. Effects sizes for the difference between heart rate retained and heart rate removed data for the subset of participants with at least one heart rate component are presented in Table 2. Paired t-tests were run to determine if the effect sizes for the datasets were significantly different, implicating biased estimates with heart rate retention. Theta power estimates were significantly greater for the heart rate retained (M = 0.71, SD = 0.23) in comparison to the heart rate removed (M = 0.51, SD = 0.16) (*t*(1,11) = 8.52, *p* < 0.001). Similarly, beta power estimates were significantly greater for the heart rate retained (M = 0.71, SD = 0.25) in comparison to the heart rate removed (M = 0.53, SD = 0.14) (*t*(1,11) = 3.92, *p* < 0.001). TBR estimates were not significantly different between the heart rate retained (M=−0.25, SD = 0.29) and the heart rate removed (M=−0.25, SD = 0.24) (*t*(1,11)=−1.28, *p* = 0.227). This pattern of results further demonstrated that theta/beta ratios are less vulnerable to noise and bias in the data relative to power values.

To determine how retention of this artifact could potentially impact associated clinical questions, we investigated the impact of heart rate retention on EEG outcomes among children in the family-based intervention vs. SAU control groups. An OLS regression was computed for effects of the intervention vs. control on TBR, with children’s age, ACE and CHAOS scores included in the model as covariates. Model output from the intention to treat analysis for the subset of children with at least one heart rate component are in Table 3 (heart rate retained) and Table 4 (heart rate removed). Comparison analyses for the complete dataset are presented in Supplementary Table S3. For the heart rate removed data, pre-intervention TBR, theta, and beta values significantly predicted post-intervention TBR, theta and beta values, but no other main effects were found (Table 4). In the heart rate retained dataset, several apparent main effects emerged, including the effects of ACEs on eyes-open theta and beta power, and the effect of PCIT treatment on eyes-closed beta power. However, none of these effects remained after proper data cleaning (Table 4), indicating that these findings were likely driven by bias and noise rather than true effects. This distinction is particularly important in clinical research, where a seemingly significant intervention effect (as reported in Table 3) could otherwise be misinterpreted as evidence for the therapy’s efficacy.

Next, we examined the impact of heart rate artifacts on children’s TBR outcomes in the intervention vs. control conditions using the per-protocol approach, partitioning intervention group children into a treatment engagers subgroup (i.e., engaged in at least one intervention session) with non-engagers omitted, via a similar OLS regression run with two (intervention-engagers vs. control group) categories. Model output from the per-protocol analysis for the subset of individuals with at least one heart rate component are in Table 5 (heart rate retained) and Table 6 (heart rate removed). Comparison analyses for the complete dataset are presented in Supplementary Table S4. Similar to the ITT model, a heart rate subset was created, and the same model was run. The heart rate-removed dataset showed similar results to the ITT model, with pre-treatment TBR, theta, and beta values significantly predicting posttreatment TBR, theta, and beta values (Table 7). In addition, there were ACE effects in the eyes-open theta (*t*(1,21) = 3.281, *p* = 0.002) and beta models *(t*(1,21) = 2.642, *p* = 0.012). The heart rate-retained model showed the same ACE effects as the heart rate removed dataset with the addition of an age effect on theta in the eyes-open condition (*t*(1,21)=−2.507, *p* = 0.021).

## Discussion

The current study investigated the impact of cardiac artifact retention on common EEG outcomes that overlap in measurement with those produced by heart rate waveforms, namely theta and beta power, and their ratio, TBR. The study was motivated by our anecdotal observation that the automated artifact screening tool ICLABEL commonly misidentifies heart rate components in pediatric, high-risk, and non-typically developing populations. Despite the successful use of heart rate artifact removal as a demonstration of ICLABEL’s accuracy in a typically developing adult sample, we found that the algorithm significantly underperforms on a pediatric dataset drawn from a sample of welfare-involved children, that may not conform to the parameters of the original training set for ICLABEL [8]. Notably we found that not only did ICLABEL misidentify heart rate in 100% of cases in our dataset, but it never provided a higher percentage composition than 5% heart rate when labeling the artifacts contributing to these components. In the majority of heart rate components, the percentage attributed to heart rate was in fact 0%, meaning that ICLABEL failed to identify heart rate as even a possible contributor to the component variance. Even more concerning were the 13 heart rate components rated by ICLABEL as primarily brain activity, meaning that even under the strictest automated artifact scan rules, these components would be retained in the final dataset. The results of this study highlight the importance of both monitoring the output of automated EEG processing pipelines and choosing processing parameters that are appropriate for the data at hand. At this time, the authors would not recommend fully automated use of EEG processing pipelines where any step was trained on primarily typically developing adult populations, including but not limited to ICLABEL, for use in pediatric data.

To improve accuracy of automated artifact detection in pediatric datasets, the parameters that differentiate these pediatric data from adult EEG, and their impact on specific overlapping artifact subtypes, must be assessed. While a full-scale investigation of this question is beyond the scope of the current paper, we suggest some possibilities based on known developmental differences in EEG characteristics. The three most prominent differences between adult and pediatric EEG data that may impact component classification are likely the slope of the 1/f distribution of EEG power, the presence of a strong individual alpha peak in the expected 8–12 Hz range, and the amount of movement artifact. These parameters are also disrupted in non-typically developing children and adults, such as in those with Fragile X Syndrome, cognitive disabilities, ADHD, and autism spectrum disorders, suggesting that similar concerns may apply to adversity-exposed children involved in the child welfare system as well [21–26]. Indeed, automated pipeline performance on children with Fragile X Syndrome and data simulated from their artifact distributions has been shown to result in inappropriate removal of brain activity when components of the pipeline are not trained on neurodevelopmental data [10]. Developmental changes have been established for slope of the 1/f distribution of EEG power, with a gradual flattening of the aperiodic component with age that occurs from infancy through early adulthood, however changes to the 1/f slope may vary in both directions (flatter or steeper) depending on the child’s age and whether the individual has a neurodevelopmental condition [27, 28]. Individual alpha peak frequency increases and plateaus in late childhood, with young children showing oscillatory (periodic) peaks in the theta range (4–7 Hz) that typically shift to the alpha range (8–12 Hz) with a pivot point in dominant frequency from theta to alpha around age 7 years [29]. Pediatric EEG data, both typically and non-typically developing, is also commonly characterized by less behavioral compliance during data collection than typically developing adult data, due to expected developmental effects on executive and motor function [30]. Movement and muscle tension artifacts can increase global EEG power and in the case of muscle tension, selectively increase high frequency power, resulting in changes to the 1/f power distribution [31]. Automated artifact identification/rejection pipelines trained primarily on adult data may struggle to disentangle theta-dominant pediatric data from theta-dominant heart rate artifact or may mischaracterize the increased beta activity in the heart rate component as a flatter, and thus more brain activity-like adult frequency distribution. Increased movement artifacts can negatively impact the quality of ICA decompositions, resulting in less clearly separated sources of activity and thus potentially more difficult automated matching of component characteristics to training sets [32]. Of note, although our data was generally consistent with the level of expected movement artifact for pediatric data, it was not overly contaminated, as evidenced by the strict minimum standards for usable data employed in this study, and the retention of 47 complete datasets based on these standards.

Our hypothesis that the theta/beta ratio (TBR) would be lower in the intervention group children relative to controls, was not supported by the appropriately cleaned (heart rate-removed) analyses. This result could occur for a number of reasons: 1) while the intervention achieves reductions in disruptive child behavior problems, it may be that TBR and associated attentional control skills are not yet impacted at posttreatment [33, 34]. 2) Further, children in this study participated in a family-based intervention that targets parenting skills. Thus, while parenting behaviors improve significantly in the intervention group of parents at post-treatment, it may be that changes to brain activity in the child may unfold more gradually in the child, as a downstream effect of experiencing more positive, responsive parenting, and well after the post-intervention assessments conducted in this study. Thus, measurements of children’s brain activity immediately after treatment may occur too soon to capture downstream child neural activity responses. Longer-term follow-up assessments of children’s brain activity following family-based interventions to determine whether such latent effects may occur. Alternatively, 3) TBR may be more associated with age-related developmental change in children’s attention regulation than any family-based intervention effects. Indeed, TBR has been posited as an indicator of attention regulation in ADHD, but that relationship has been called into question recently by indications that TBR measurement is strongly impacted by developmental shifts in EEG, including the theta to alpha dominance shift [35, 36].

Importantly, in our data with heart rate retained, TBR results were largely unaffected in terms of bias and Cohen’s D, likely due to increases in both theta and beta power, leading to relatively small changes in the ratio between the two frequencies. We found that the heart rate-retained data displayed a spurious intervention effect in eyes-closed beta power and spurious ACEs effect on both eyes open theta and beta power, with the heart rate artifact retained in the data. Further, increased bias was observed in the effect sizes of difference from zero for both theta and beta power estimates, suggesting increased noise in the heart rate-retained data that increased risk of both false positive and negative conclusions, depending on which variables of interest may be confounded with the presence or absence of heart rate artifact.

One question that remains from this work is whether other artifact subtypes such as blinks and saccadic eye movements are similarly affected by the concerns described above. Given the focus on TBR and its component power structure as the outcome of interest in this study, and its relevance to hypothesized neural effects of adversity and intervention, this study focused on these overlapping artifacts alone. The higher amplitude nature of most blinks and saccadic eye movements, relative to brain activity amplitudes, may also allow for simpler amplitude thresholding as an artifact removal strategy and may thus lend themselves to easier automated identification and removal. However, studies with neural outcome measure in the delta/low theta frequency range, or in far frontal cortices, that overlap with dominant frequency components and spatial distribution of blinks and saccadic eye movements, or studies where the relative amplitude difference between eye artifacts and expected brain activity is smaller, may wish to consider the possibility of similar concerns on automated artifact identification in pediatric datasets.

This study is limited to investigation of only a few EEG outcomes and their relevance to heart rate artifacts only, based on the appropriateness of the experimental protocol in which the data was collected, and the anecdotal observation by the authors and our colleagues of ICLABEL mislabeling most frequently for heart rate across pediatric, high-risk, and neurodevelopmental datasets. Future work focused on datasets with relevant outcomes overlapping with other types of artifacts, such as blinks, saccadic eye movements, and muscle activity, should investigate whether similar impacts are observed for these artifacts as well. Our investigation was also limited to resting EEG in a small sample of pediatric data, from children ages 3–7 years, so may not generalize to younger or older children or evoked tasks. However, these should still apply to a more extended age range of pediatric data. Sample sizes for the subgroup of individuals with at least one heart rate component was relatively small, however this concern is partially mitigated by the primary comparison of interest as the within-subject comparisons for bias, and the similarity in effects observed in the cleaned (heart rate removed) results between the subgroup and the full sample.

Finally, this methodological investigation of heart rate artifact impacts on pediatric EEG measures revealed increased statistical bias in the data and a spurious intervention effect on TBR outcomes. We encourage researchers to carefully examine their EEG data for residual heart rate artifacts when interpreting theta and beta power, or TBR. Most importantly, we uncovered strong empirical evidence for inaccurate heart rate identification in pediatric data by the ICLABEL algorithm, which should serve as a warning for any researchers using fully automated pipelines to carefully examine the appropriateness of the training set for that pipeline as it compares to their own data. Despite an expectation of some mislabeling from anecdotal observations, we were surprised by the 100% miss rate for ICLABEL on heart rate in our data. We advocate for additional development of large-scale training sets specific to pediatric, high-risk, and neurodevelopmental EEG populations, prior to use of automated pipelines for unsupervised artifact identification and rejection. The purpose of automated pipelines is not only to reduce workload but to increase standardization and reduce human bias in EEG data processing, however that purpose is not yet fully realized given that a “one-size-fits-all” approach cannot be implemented on populations with different inherent characteristics to both brain activity and behavior during data collection. Given the sensitivity of ICA to unique variations in data composition and quality, we highly recommend supervision and careful scrutiny of all automated processing results using ICA to increase understanding of and confidence in unbiased experimental results.

## Methods

### Sample

Data were drawn from a larger randomized controlled parallel group superiority trial investigating the effectiveness of a family-based intervention for child-welfare involved families (start date 02/18/2016; #NCT02684903. Funding: NIDA R01 036533). The full sample consisted of *N* = 204 families referred by child welfare, who were enrolled, completed baseline assessments, and randomized to the intervention or services-as-usual control conditions. Information on recruitment, screening procedures, and clinical trial design can be found [37]. The parent study received ethics approval from the University of Oregon Institutional Review Board (IRB #07102014) the State of Oregon’s Department of Health and Human Services. All methods were performed in accordance with relevant guidelines and regulations and adhered to the principles of the Declaration of Helsinki. No adverse effects were reported for this study.

Parents and guardians provided written informed consent to participate, and children gave verbal assent prior to engaging in any study procedures. Families were allowed to withdrawal from the trial at any time. Children were between 3 and 7 years old (M = 5.13, SD = 1.45 years) at study entry, and 53.2% male, 46.8% female. Parents were at least 18 + years of age, and a majority (78.5%) of families were living below the poverty line, based on the U.S. Department of Health and Human Services guidelines for 2020. Children exposed to sexual abuse were excluded from the study due to compatibility with the treatment being studied. Full demographics are located in Supplementary Table S1. No statistically different differences were observed across conditions on child age, sex, parent age, and household income.

## Procedures

Children and their parents participated in pre- and post-treatment assessments, completed in two separate lab visits scheduled approximately one week apart. Procedures relevant to the current study are described here. Children’s resting state EEG was collected during the second laboratory visit at baseline (pre) and again at post-intervention. Parents completed a variety of questionnaires, while children were taken to a separate testing room and fitted for simultaneous electroencephalogram (EEG) and electrocardiogram (ECG) recordings, before completing a 4-minute resting EEG task, a 3-minute resting baseline measure of cardiac physiology, and two executive function tasks during which simultaneous EEG and cardiac physiology were recorded. Parents completed their EEG assessments in the previous visit, during which their child observed the sensor net fittings to help desensitize them to the process. Parent questionnaires were completed in an interview-like format with a trained researcher. Please see [37] for a description of the full study protocol. Following pre-intervention assessments, families were randomized to condition. Identical assessments were conducted at post-treatment and on the same timeline across both conditions, on average at 7.84 months post-study entry (*SD* = 2.34).

### Resting-State EEG.

Children completed a resting state EEG at the pre-treatment assessment and again at post-treatment. Children then sat in the acquisition room with a trained and familiar research assistant. Research assistants used a standardized script to ensure all participants were provided with the same instructions. The resting EEG task required children to complete 1-minute alternating eyes-closed (EC) and eyes-open (EO) conditions for a total of 4 minutes, while seated in the dark room, while a familiar research assistant sat nearby. Children were instructed to close their eyes during the eyes-closed (EC) condition, and during the eyes-open (EO) condition, to fixate on a blank screen. Previous work has found significant variation in amplitude and frequency depending on the EC or EO task condition [38, 39].

## Survey Measures

### The Adverse Childhood Experiences Scale.

Parents reported on their child’s exposure to adverse childhood experiences using the Adverse Childhood Experiences Scale (ACES) at pre-treatment [40]. Items reflect parental substance abuse, parental divorce, domestic violence exposure, parental incarceration, parental mental health, and abuse. Each item was coded 0 or 1 for the absence or presence of each risk factor, respectively, and summed to create a composite risk score ranging from 0 to 12. A higher value indicated more experience with childhood trauma.

### Confusion, Hubbub, and Order Scale.

Parents completed the Confusion, Hubbub, and Order Scale (CHAOS) at pre-treatment, a 14-item binary scale that measures an individual’s home environment in terms of processes that are distinct from sociodemographic measures [41]. Examples include noise, crowding, and overall commotion in the home setting. Each item is coded 0 or 1 for the absence or presence of household characteristics, respectively. Scores ranged from 0 to 14, with higher scores indicating greater household chaos.

### Behavioral Parenting Intervention condition.

Children who were randomized to the intervention condition received a family-based intervention, that employs live coaching of parent–child interactions to strengthen positive parenting and improve disruptive child behavior problems [42]. *Services-as-Usual Control condition*. The family services-as-usual (SAU) control condition is an ethical comparison group in which families receive typically delivered services provided by child welfare agencies (e.g., school-based services, in-home family visitation, supplemental nutrition assistance, etc.).

#### Missing Data

Missing data in children’s resting EEG scores were evaluated using missing values analyses. Results show that 34.3% and 45.6% of children’s resting EEG data was missing at pre-treatment and post-treatment, respectively. Missingness at pre-treatment was primarily due to difficulty placing the EEG cap on participants or participant refusal. Missingness at post-treatment was primarily due to children not completing the post-treatment assessment entirely (*n* = 51) or participant refusal (*n* = 17). Proportions of missingness at both pre- and post-treatment differed significantly by age (*χ*2 (30) = 48.97, *p* = .016, *χ*2 (5) = 19.01, *p* = .002), with younger children producing more missing data than older children. 67% of missing EEG data at pre-treatment and 69% of missing EEG data at post-treatment were in children ages 3–4 at baseline. We did not find evidence of differential missingness across the randomization groups. Further, we limited the analysis to complete cases (i.e., observations with both pre- and post-measurements), an approach may limit the generalizability of our findings (e.g., due to the underrepresentation of younger children), but we prioritized the analytic simplicity of this tradeoff.

## EEG Pre-Processing

All children were fitted with a 64-channel EGI Hydrocel Geodesic Sensor Net (EGI Philips; Eugene, OR) except if the child’s cap was too small, whereby a high-density 256-channel net was used (*n* = 9). For these participants, EEG information was mapped onto the original 64-net to match. EEG was continuously recorded at a sampling rate of 500 Hz with 0.1 Hz high-pass filter using the EGI Net and Net Amp 300 amplifier integrated with Net Station software version 5.2.0.2 (EGI Philips; Eugene, OR), with sensors placed according to the International 10–20 system. After the net was placed, impedance was measured and corrected where possible. Data was recorded and filtered from 1 to 80 Hz and referenced to Cz. Raw data files were reviewed offline. Bad sensors were interpolated using the spherical spline interpolation (no more than 5% of sensors were removed for bad data). Data segments with an excessive amount of artifacts (large movements or electrical spiking) were manually removed before individual component removal (ICA). ICA was implemented using EEGLAB in Matlab 2021.0 software [12]. was decomposed into 64 components; ICA components were examined and removed manually if they contained primarily artifact, and data was re-referenced to an average reference. Lastly, data was then segmented into 2-second epochs, and epochs with an amplitude exceeding 120 μV were removed prior to analysis.

Out of the full sample of 204, 47 children had complete case data (i.e., both pre and post) and were used in the analyses below. The majority of the data removed was due to a lack of complete data, rather than messiness. Only 10 files were removed from analyses due to excessive artifact. Usability of data was determined by a trained individual and based on the number of channels removed, clean trials, and number of ICA components retained. If the number of channels requiring interpolation was greater than 5% (or 3 channels), the number of clean trials was below 50% of the total trial count, or less than 40% of ICA components were retained, files were excluded from analyses. During the final post-ICA segmentation and amplitude thresholding, the average number of epochs removed per file was (M = 104.96, SD = 101.18) out of a total (M = 624.29, SD = 216.63) number of epochs, or 83% retained, suggesting that the data were not unusually artifact-ridden and thus performance of ICLABEL was unlikely to be due to excessive noise in the dataset. These means were consistent across conditions.

## Power Calculation

Power in theta (4–7 Hz), alpha (7.5–12.5 Hz), and beta (13–30 Hz) were computed, with boundaries for the frequency bands were defined based on individual alpha peak frequency (IAF). Anchoring frequency bands to IAFs can more accurately capture age-related changes and reduces the contribution of slow alpha activity to the frequency estimates [43]. IAFs were calculated by identifying the maximum attenuation of power within the 6–16 Hz range from the difference in posterior power during eyes-closed and eyes-open tasks [44]. Frequency bands were then anchored to IAFs [43, 45] using the equations (1–3) below:

Theta(1):0.4⋆IAF-0.8⋆IAF

Beta(2):1.2⋆IAF-30Hz

Alpha(3):0.8⋆IAF-1.2⋆IAF

Relative power was calculated using the *pwelch* function in MATLAB 2021.0b. Relative power is preferred over absolute power as it can account for differences in skull thickness, as it changes across age in development [46]. The channels of interest for this analysis were the channels located in the frontal region: 2, 3, 6, 8, 9, 11, 12, 13, 14, 57, 59, 60 (Supplementary Figure S1, in red). Theta-beta ratios were calculated by taking the frontal relative power of theta divided by the frontal relative power of beta for a given participant, task, and time. Theta-beta ratios were partially skewed, thus, values were log transformed before being used in any analyses.

### Heart Rate Artifact Identification

Heart rate artifacts were identified by examining all components in the post-ICA step of data processing. Components were opened and each portion of the components (topography, activity scroll, continuous data, and power spectrum) were examined for patterns that typically distinguish heart rate artifacts from other artifacts. Patterns that were specifically looked for were a dipole over the head in the topography, evenly spaced peaks on the activity scroll, tiny and evenly spaced dots on the continuous data, and an unusual power spectrum shape (see Fig. 1 for example). If the component appeared to have at least two of these characteristics, it was classified as a heart rate component and not removed from the data. All other artifacts were removed as normal.

To further explore the effect of heart rate artifact on pediatric EEG data, ICLABEL percentages for each category (brain, muscle, eye, channel noise, line noise, and other) were acquired for each heart rate component. A count was created each time a category had the highest ICLABEL percentage for a component to determine what type of artifact heart rate is most often misidentified as (Fig. 2).

### Data Analysis

Data analysis methods were conducted to explore the impact of heart rate artifacts when left in otherwise cleaned data. Regression models were taken from [37]. All analyses were conducted in R. Only participants who had complete case data (i.e., both pre and post) were used in the following analyses (*n* = 47).

### Heart Rate Effect Analyses

In order to determine the effect that heart rate artifact has on the data, power, and subsequent analyses, binary variables were created indicating whether there was at least one heart rate component in pre and post-data for each participant. To explore the impact on power values, ANOVA was run for eyes-closed and eyes-open data, with heart rate during pre or post, run separately, as the dependent variable. Theta-beta, theta, and beta values were used as the outcomes and ran separately. Models including ACE binary score were also run. ACE binary scores were operationalized as 0 for low ACE scores in which participants had exposure to 3 or less ACEs and 1 for high ACE which indicated that participants had exposure to 4 or more ACEs, an acceptable way of grouping ACEs [47, 48]. Level of PCIT engagement was also included in these models. The assumptions of ANOVA (independence, normality homogeneity, and outliers) were explored in R and were not violated within this dataset.

Correlations were run to explore the relationship between the presence of heart rate and theta-beta ratios, theta and beta power values. Lastly, bias was explored between the heart rate, and no heart rate means for theta-beta ratios, and theta and beta power. To quantify potential bias in the outcome measures, a difference score was calculated for each participant at pre and post, and for eyes-closed and eyes-open tasks. An example for pre-treatment data is below:

$${\text{Bias}}_{pre}=\beta_{pre\;HR\;\text{data}}-\beta_{\text{post}\;HR\;\text{data}}$$

This difference score represents the deviation introduced by the contamination of the EEG data by heart rate artifact. If heart rate has no biasing effect on the measures, the mean difference score across participants would be expected to be zero. Conversely, a mean difference score that is significantly different from zero would indicate bias in the outcome measure when heart rate artifact is present in the data. For each combination of task (EC/EO), Cohen’s D was calculated to estimate the magnitude of bias at both pre and post timepoints relative to zero, with a CI of 95%. A Cohen’s D significantly different from zero indicates that heart rate artifact introduces a shift in the outcome measures, with the magnitude of the effect size reflecting the breadth of this biasing effect in standardized units. Calculations were performed in R using the *effectsize* package and all variables were normally distributed.

### Testing Heart Rate Artifact Differences in TBR Outcomes

Intention to treat (ITT) superiority analyses were conducted to test the impact of intervention vs. control conditions on children’s heart-rate retained and heart-rate removed theta/beta ratio (TBR) outcomes. OLS regression was used to examine the intervention (treatment vs. control) and the resting EEG task conditions (EC/EO) on post-treatment theta/beta ratio scores, while controlling for age and pre-treatment theta/beta ratio. Moderating effects of ACES and CHAOS scores were also evaluated within the model. ITT analyses were run on the dataset (n = 47) with participants that had both heart rate and no heart rate in their data, as well as for the heart rate only participant subset (n = 27) to directly compare the two. Next, to account for the conservative treatment effect estimates in ITT analysis, a per-protocol analysis also was performed on control group participants (n = 23) and those who engaged in at least one intervention session (n = 24). Identical regression analyses to the ITT analyses were run. Per-protocol analyses were run on the dataset (n = 47) with participants that had both heart rate and no heart rate in their data, as well as for the heart rate only participant subset (n = 27) to directly compare the two.

## Supplementary Material

Supplementary Files

This is a list of supplementary files associated with this preprint. Click to download.

- SupplementaryInformation.docx

## Figures and Tables

**Table 1:** Cohen’s D of Variables of Interest for Full Dataset

Complete Cases	Control (n = 23)	Tx Non-Engagers (n = 6)	Tx Engagers (n = 18)
TBR			
EC-Pre	−0.03 [−0.44, 0.38]	−0.41 [−1.23, 0.45]	−0.07 [−0.28, 0.39]
EC-Post	−0.46 [−0.89, 0.03]	−0.28 [−1.09, 0.55]	−0.40 [−0.87, 0.09]
EO-Pre	0.20 [−0.22, 0.61]	−0.53 [−1.37, 0.35]	0.18 [−0.28, 0.65]
EO-Post	−0.34 [−0.75, 0.09]	−0.31 [−1.11, 0.53]	−0.17 [−0.63, 0.30]
Theta			
EC-Pre	0.48 [0.04, 0.90]	0.62 [−0.29, 1.47]	0.41 [−0.08, 0.89]
EC-Post	0.20 [−0.23, 0.60]	0.65 [−0.26, 1.52]	0.62 [0.11, 1.12]
EO-Pre	0.55 [0.11, 0.98]	0.36 [−0.48, 1.17]	0.42 [−0.07, 0.90]
EO-Post	0.46 [0.03, 0.89]	0.65 [−0.26, 1.52]	0.55 [0.04, 1.04]
Beta			
EC-Pre	0.46 [0.03, 0.89]	0.59 [−0.30, 1.45]	0.34 [−0.14, 0.81]
EC-Post	0.55 [0.09, 0.97]	0.69 [−0.24, 1.57]	0.70 [0.17, 1.21]
EO-Pre	0.50 [0.06, 0.93]	0.39 [−0.46, 1.21]	0.32 [−0.14, 0.81]
EO-Post	0.55 [0.10, 0.98]	0.74 [−0.20, 1.63]	0.59 [0.08, 1.09]

*Note:* TBR= Theta-Beta Ratio, EC= Eyes-Closed Rest, EO= Eyes-Open Rest

**Table 2:** Cohen’s D of Variables of Interest for Heart Rate Subset

Complete Cases	Control (n = 14)	Tx Non-Engagers (n = 4)	Tx Engagers (n = 9)
TBR			
EC-Pre	−0.04 [−0.58, 0.50]	−0.50 [−1.52, 0.58]	−0.09 [−0.71, 0.53]
EC-Post	−0.41 [−0.95, 0.14]	−0.34 [−1.33, 0.70]	−0.46 [−1.10, 0.20]
EO-Pre	0.26 [−0.30, 0.81]	−0.68 [−1.75, 0.47]	0.25 [−0.41, 0.92]
EO-Post	−0.27 [−0.80, 0.27]	−0.37 [−1.33, 0.70]	−0.31 [−0.97, 0.37]
Theta			
EC-Pre	0.66 [0.07, 1.30]	0.82 [−0.39, 1.93]	0.58 [−0.11, 1.24]
EC-Post	0.35 [−0.19, 0.89]	0.88 [−0.35, 2.02]	0.84 [0.09, 1.55]
EO-Pre	0.78 [0.17, 1.37]	0.44 [−0.63, 1.44]	0.64 [−0.09, 1.34]
EO-Post	0.57 [−0.01, 1.12]	1.22 [−0.17, 2.53]	0.80 [0.02, 1.53]
Beta			
EC-Pre	0.66 [0.04, 1.25]	0.78 [−0.41, 1.88]	0.47 [−0.19, 1.11]
EC-Post	0.46 [−0.12, 1.03]	0.95 [−0.31, 2.13]	0.98 [0.20, 1.72]
EO-Pre	0.73 [0.10, 1.33]	0.47 [−0.60, 1.49]	0.46 [−0.24, 1.14]
EO-Post	0.43 [−0.15, 0.98]	1.04 [−0.26, 2.27]	1.07 [0.22, 1.89]

*Note:* TBR= Theta-Beta Ratio, EC= Eyes-Closed Rest, EO= Eyes-Open Rest

**Table 3:** Model Output for ITT for Heart Rate Retained Subset (n = 27)

Predictor	Estimate	Standard Error	t-value	p-value
*Eyes Closed-TBR*				
Intercept	0.229	0.216	1.064	0.300
**Pre TB Ratio**	**0.696**	**0.155**	**4.495**	**0.000**
Intervention	−0.031	0.021	−1.471	0.157
Age	−0.007	0.016	−0.412	0.684
ACE	0.005	0.012	0.380	0.708
CHAOS	0.009	0.006	1.417	0.172
*Eyes Open-TBR*				
Intercept	0.313	0.170	1.844	0.081
**Pre TB Ratio**	**0.680**	**0.131**	**5.184**	**0.000**
Intervention	0.002	0.020	0.077	0.940
Age	−0.019	0.014	−1.412	0.174
ACE	0.010	0.011	0.931	0.364
CHAOS	0.005	0.006	0.921	0.369
*Eyes Closed- Theta*				
Intercept	0.006	0.013	0.443	0.662
**Pre Theta**	**0.575**	**0.201**	**2.859**	**0.009**
Intervention	0.001	0.002	0.386	0.704
Age	0.000	0.001	0.042	0.967
ACE	0.001	0.001	1.287	0.212
CHAOS	0.000	0.000	1.020	0.320
*Eyes Open- Theta*				
**Intercept**	**0.021**	**0.010**	**2.094**	**0.049**
**Pre Theta**	**0.527**	**0.177**	**2.969**	**0.008**
Intervention	0.002	0.002	1.360	0.189
**Age**	**−0.003**	**0.001**	**−2.568**	**0.018**
**ACE**	**0.003**	**0.001**	**2.745**	**0.012**
CHAOS	0.000	0.000	0.627	0.538
*Eyes Closed- Beta*				
**Intercept**	**0.002**	**0.001**	**2.325**	**0.030**
**Pre Beta**	**0.367**	**0.130**	**2.817**	**0.010**
Intervention	**0.000**	**0.000**	**2.153**	**0.043**
Age	0.000	0.001	1.250	0.225
ACE	0.000	0.001	1.448	0.162
CHAOS	0.000	0.000	−1.019	0.320
*Eyes Open- Beta*				
**Intercept**	**0.002**	**0.001**	**2.511**	**0.021**
**Pre Beta**	**0.363**	**0.147**	**2.463**	**0.023**
Intervention	0.000	0.000	1.364	0.188
Age	0.000	0.000	−0.120	0.905
**ACE**	**0.000**	**0.000**	**2.762**	**0.012**
CHAOS	0.000	0.000	−0.984	0.337

**Table 4:** Model Output for ITT for Heart Rate Removed Subset (n = 27)

Predictor	Estimate	Standard Error	t-value	p-value
*Eyes Closed-TBR*				
Intercept	0.117	0.185	0.631	0.535
**Pre TB Ratio**	**0.754**	**0.131**	**5.744**	**0.000**
Intervention	−0.022	0.020	−1.115	0.278
Age	0.011	0.014	0.737	0.469
ACE	0.018	0.011	1.588	0.127
CHAOS	−0.003	0.006	−0.539	0.595
*Eyes Open-TBR*				
Intercept	0.246	0.143	1.715	0.102
**Pre TB Ratio**	**0.751**	**0.109**	**6.891**	**0.000**
Intervention	0.017	0.019	0.914	0.372
Age	−0.006	0.013	−0.489	0.630
ACE	0.005	0.011	0.507	0.618
CHAOS	0.001	0.005	0.151	0.882
*Eyes Closed- Theta*				
Intercept	0.000	0.012	0.016	0.988
**Pre Theta**	**0.609**	**0.188**	**3.243**	**0.004**
Intervention	−0.001	0.001	−0.730	0.474
Age	0.001	0.001	1.141	0.267
ACE	0.001	0.001	1.267	0.219
CHAOS	0.000	0.000	0.289	0.775
*Eyes Open- Theta*				
**Intercept**	**0.015**	**0.006**	**2.522**	**0.020**
**Pre Theta**	**0.632**	**0.108**	**5.829**	**0.000**
Intervention	0.001	0.001	0.998	0.330
Age	−0.001	0.001	−1.952	0.065
ACE	0.001	0.001	1.227	0.234
CHAOS	0.000	0.000	1.146	0.265
*Eyes Closed- Beta*				
Intercept	0.001	0.001	1.355	0.190
**Pre Beta**	**0.602**	**0.122**	**4.938**	**0.000**
Intervention	0.000	0.000	0.735	0.471
Age	0.000	0.000	1.042	0.309
ACE	0.000	0.000	−0.136	0.893
CHAOS	0.000	0.000	0.669	0.512
*Eyes Open- Beta*				
Intercept	0.001	0.001	1.249	0.226
**Pre Beta**	**0.771**	**0.140**	**5.520**	**0.000**
Intervention	0.000	0.000	−0.437	0.667
Age	0.000	0.000	−0.915	0.371
ACE	0.000	0.000	1.280	0.215
CHAOS	0.000	0.000	0.670	0.511

**Table 5:** Model Output for Per-Protocol for Heart Rate Subset (n = 27)

Predictor	Estimate	Standard Error	t-value	p-value
*Eyes Closed*				
Intercept	0.260	0.220	1.185	0.250
**Pre TBR**	**0.696**	**0.157**	**4.428**	**0.000**
Intervention-Engagers	−0.048	0.039	−1.219	0.237
Age	−0.006	0.016	−0.393	0.698
ACE	0.003	0.012	0.274	0.787
CHAOS	0.009	0.007	1.412	0.173
*Eyes Open*				
Intercept	0.305	0.171	1.787	0.090
**Pre TBR**	**0.681**	**0.131**	**5.213**	**0.000**
Intervention-Engagers	0.012	0.035	0.328	0.746
Age	−0.019	0.014	−1.346	0.194
ACE	0.010	0.011	0.881	0.389
CHAOS	0.005	0.006	0.863	0.399
*Eyes Closed- Theta*				
Intercept	0.004	0.013	0.337	0.739
**Pre Theta**	**0.576**	**0.190**	**2.900**	**0.009**
Intervention-Engagers	0.002	0.003	0.833	0.414
Age	0.000	0.001	0.164	0.871
ACE	0.001	0.001	1.220	0.236
CHAOS	0.000	0.000	0.911	0.373
*Eyes Open- Theta*				
Intercept	0.019	0.010	1.839	0.081
**Pre Theta**	**0.513**	**0.178**	**2.878**	**0.009**
Intervention-Engagers	0.003	0.003	1.167	0.257
**Age**	**−0.003**	**0.001**	**−2.507**	**0.021**
**ACE**	**0.003**	**0.001**	**2.748**	**0.012**
CHAOS	0.000	0.000	0.591	0.561
*Eyes Closed- Beta*				
Intercept	0.001	0.001	1.823	0.083
**Pre Beta**	**0.356**	**0.127**	**2.808**	**0.011**
Intervention-Engagers	0.001	0.000	2.424	0.024
Age	0.000	0.000	1.417	0.171
ACE	0.001	0.000	1.483	0.153
CHAOS	0.000	0.000	−1.092	0.247
*Eyes Open- Beta*				
**Intercept**	**0.002**	**0.001**	**2.150**	**0.044**
**Pre Beta**	**0.354**	**0.150**	**2.363**	**0.028**
Intervention-Engagers	0.000	0.000	1.045	0.309
Age	0.000	0.000	−0.110	0.914
**ACE**	**0.000**	**0.000**	**2.788**	**0.011**
CHAOS	0.000	0.000	−0.967	0.345

**Table 6:** Model Output for Per-Protocol Cleaned Heart Rate Subset (n = 27)

Predictor	Estimate	Standard Error	t-value	p-value
*Eyes Closed*				
Intercept	0.134	0.189	0.711	0.485
**Pre TBR**	**0.757**	**0.133**	**5.708**	**0.000**
Intervention-Engagers	−0.032	0.036	−0.882	0.388
Age	0.011	0.015	0.755	0.459
ACE	0.017	0.011	1.496	0.149
CHAOS	−0.003	0.006	−0.525	0.605
*Eyes Open*				
Intercept	0.227	0.144	1.578	0.130
**Pre TBR**	**0.747**	**0.108**	**6.935**	**0.000**
Intervention-Engagers	0.036	0.033	1.077	0.294
Age	−0.005	0.013	−0.426	0.675
ACE	0.005	0.010	0.490	0.630
CHAOS	0.000	0.005	0.075	0.941
*Eyes Closed- Theta*				
Intercept	0.001	0.012	0.120	0.906
**Pre Theta**	**0.598**	**0.189**	**3.163**	**0.005**
Intervention-Engagers	−0.001	0.002	−0.332	0.743
Age	0.001	0.001	1.148	0.264
ACE	0.001	0.001	1.132	0.270
CHAOS	0.000	0.000	0.253	0.803
*Eyes Open- Theta*				
Intercept	0.010	0.007	1.416	0.166
**Pre Theta**	**0.594**	**0.137**	**4.340**	**0.000**
Intervention-Engagers	0.002	0.002	0.729	0.471
Age	−0.001	0.001	−1.347	0.187
**ACE**	**0.002**	**0.001**	**3.281**	**0.002**
CHAOS	0.000	0.000	0.799	0.430
*Eyes Closed- Beta*				
Intercept	0.001	0.001	1.195	0.245
**Pre Beta**	**0.601**	**0.121**	**4.961**	**0.000**
Intervention-Engagers	0.000	0.000	0.901	0.378
Age	0.000	0.000	1.108	0.280
ACE	0.000	0.000	−0.159	0.875
CHAOS	0.000	0.000	0.593	0.559
*Eyes Open- Beta*				
**Intercept**	**0.002**	**0.001**	**2.999**	**0.005**
**Pre Beta**	**0.254**	**0.121**	**2.099**	**0.043**
Intervention-Engagers	0.000	0.000	0.668	0.509
Age	0.000	0.000	0.488	0.629
**ACE**	**0.000**	**0.000**	**2.642**	**0.012**
CHAOS	0.000	0.000	−0.421	0.677

## Data Availability

Full clinical trial protocol and study information can be accessed here-https://clinicaltrials.gov/study/NCT02684903?locStr=Eugene,%20OR&country=US&state=Oregon&city=Eugene&intr=PCIT&viewType=Card&rank=1. Cleaned datasets used and/or analyzed during the current study are available from the corresponding author on reasonable request. Full R code and outputs are available on GitHub-https://github.com/brennaarledge-debug/CAPS_Psychophys26.git.
